# Assessing the Correlation Between Visual Acuity and Critical Fusion Frequency in Acute Optic Neuritis Before and After Steroid Therapy

**DOI:** 10.7759/cureus.49965

**Published:** 2023-12-05

**Authors:** Ryo Tsumura, Yosuke Harada, Hideki Chuman, Yoshiaki Kiuchi

**Affiliations:** 1 Department of Ophthalmology, Hiroshima University, Hiroshima, JPN; 2 Department of Ophthalmology, Faculty of Medicine, University of Miyazaki Hospital, Miyazaki, JPN

**Keywords:** steroid pulse therapy, anti-mog antibodies, anti-aqp4 antibody, critical fusion frequency, acute optic neuritis

## Abstract

Background

Optic nerve diseases include inflammatory optic nerve diseases such as vasculitis, metabolic optic neuropathy, ischemic optic neuropathy, and optic neuritis. In this study, patients with acute optic neuritis are classified with better and poor visual acuity based on visual acuity after one month of steroid pulse therapy. To determine prognosis, initial visual acuity and critical fusion frequency (CFF) values will be compared with those recorded one month after treatment and at the last visit.

Methods

Visual acuity and CFF were evaluated one month after the start of treatment in patients diagnosed with acute optic neuritis, and follow-up was available for at least three months at Hiroshima University Hospital.

Results

All patients received steroid pulse therapy as initial treatment. After one month of treatment, visual acuity and CFF at the last visit were significantly improved in the group with improved visual acuity compared to the group with impaired visual acuity.

Conclusions

Visual acuity at the initial visit did not affect treatment outcome, and final visual acuity and CFF after one month of treatment for acute optic neuritis were better in patients with better visual acuity. Therefore, visual acuity values one month after treatment initiation may affect treatment outcomes.

## Introduction

Optic nerve diseases include inflammatory optic nerve diseases such as vasculitis, metabolic optic neuropathy, ischemic optic neuropathy, and optic neuritis. Among these, acute optic neuritis is a disease that causes optic nerve inflammation and rapid vision loss. The Optic Neuritis Treatment Trial (ONTT), a treatment trial conducted in the U.S., reported that steroid pulse therapy was associated with a significantly short time to visual function recovery. However, the one-year visual acuity did not differ substantially between the steroid pulse group and the placebo group [1]. Acute optic neuritis has recently been classified into several disease types, including sclerosis, neuromyelitis optica spectrum disorder, and anti-MOG-related disorders. Steroid pulse therapy is used as the initial treatment in both cases [2].

There are several reports on the prognostic factors of optic neuritis. Optic neuritis with positivity to antibodies, such as anti-MOG antibodies and anti-aquaporin-4 antibodies, may have a poor visual prognosis. [2,3] Classification based on antibody positivity can facilitate prognostic prediction and optimize acute treatment and preventive therapy [2]. Previous reports have described factors associated with poor prognosis, including poor visual acuity at initial presentation [4,5], advanced age at onset [6], and long lesions in the optic nerve on MRI at initial presentation [7]. However, these are all clinical features in the early stages of the disease, and there is limited information on the prognostic factors of post-steroid pulse treatment. In Japan, patients with optic neuritis were closely monitored based on changes in visual acuity, visual field, and critical fusion frequency (CFF) after treatment. The CFF is a sensitive, rapid, and simple test for detecting abnormal optic nerve function. A CFF of >35 Hz and <25 Hz are considered normal and abnormal, respectively [7]. Unlike visual field testing, CFF, similar to visual acuity, can provide immediate results. Therefore, it helps evaluate optic neuritis after treatment.

In Japan, patients with optic neuritis were closely monitored based on changes in visual acuity, visual field, and CFF after treatment. The CFF is a sensitive, rapid, and simple test for detecting abnormal optic nerve function. A CFF of >35 Hz and <25 Hz are considered normal and abnormal, respectively [8]. Unlike visual field testing, CFF, similar to visual acuity, can provide immediate results. Therefore, it helps evaluate optic neuritis after treatment.

The current study aimed to determine the use of visual acuity and CFF assessment at one month after treatment initiation, which is considered the appropriate time to determine the efficacy of steroid pulse therapy and to subsequently administer oral steroids for acute optic neuritis, a prognostic factor of optic neuritis.

## Materials and methods

Data on sex, age at onset, visual acuity, slit-lamp microscopy, CFF, fundus examination findings, and orbital MRI results were collected from the medical records of the patients. The results were also collected if anti-aquaporin-4 antibody and anti-myelin oligodendrocyte glycoprotein antibody concentrations were measured. The clinical features of acute optic neuritis were examined using visual acuity one month after the start of steroid pulse therapy and visual prognosis based on CFF.

Exclusion criteria

Optic nerve diseases other than inflammatory optic nerve symptoms, cases in which steroid pulses were not performed, and cases in which the patient could not be followed for three months.

Classification of disease type

Patients with visual acuity below 0 LogMAR at one month after treatment were classified into the better visual acuity group and the poor visual acuity group. The visual acuity one month after treatment was compared to assess the transition and prognosis of visual acuity and CFF values. A CFF of >35 Hz indicated good acuity and a CFF of <35 Hz represented poor acuity.

Treatment methods

Steroid pulse therapy (methylprednisolone 1000 mg) was administered for three days. The attending physician decided whether or not to administer post-therapy with oral prednisolone. Patients who did not respond to treatment received at least two steroid pulse therapies at the physician’s discretion. Patients who did not respond to steroid pulse therapy and those with indications for plasma exchange were treated with plasma exchange.

Visual acuity and CFF measurements

Visual acuity was expressed as LogMAR. Exponential valves were converted to 2.0 logMAR, manual valves to 2.3 logMAR, photic valves to 3.0 logMAR, and no photic valves to 3.1 logMAR. CFF measurements were performed using the Kindai Type II Flicker Value Analyzer (Yagami).

Antibody concentrations and MRI findings

Anti-AQP4 antibody measurements were performed using the cell-based assay (CBA) or the enzyme-linked immune sorbent assay (ELISA). CBA was used for anti-MOG antibodies. Antibody positivity was defined as positivity for each antibody, and antibody negativity as negativity for each antibody.

Moreover, orbital MRI was performed to evaluate optic nerve lesion length on fat-suppressed T2-weighted or gadolinium contrast-enhanced T1-weighted images. Long optic nerve lesions on MRI were defined as lesions over half of the total optic nerve length.

Statistical analysis

Between the two independent groups, the Mann-Whitney U test was used to test differences in median values for each item, such as age. The chi-square was used to test for proportions for each item, such as sex.

## Results

The study included 59 patients (22 men and 37 women), with an average age of 46.0 ± 20.8 years. The average observation period was 85.9 ± 95.3 weeks. The better vision group included 29 patients (11 men and 18 women), and the poor vision group comprised 30 patients (11 men and 19 women).

**Figure 1:**
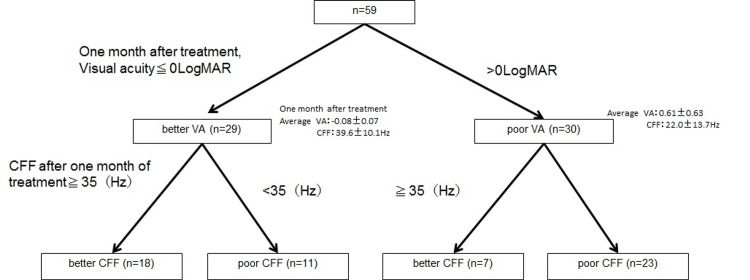
Classification methods

The mean visual acuity of the better vision group at one month after treatment was −0.08 ± 0.07, and their CFF was 39.6 ± 10.1 Hz. The mean visual acuity of the poor vision group at one month after treatment was 0.61 ± 0.63, and their CFF was 22.0 ± 13.7 Hz. Two patients in the better vision group and eight in the poor vision group presented with recurrence (Figure 1).

The presence or absence of each antibody did not significantly differ between the poor vision group and the better vision group (chi-square, p = 0.32). Moreover, the lesion length on MRI did not significantly differ between the two groups (chi-square, p = 0.41).

The mean age at onset was 38.9 ± 18.6 years in the better vision group. In particular, some participants were aged 5-70 years, and five children were aged under 15 years. The mean age at onset was 52.0 ± 21.1 years in the poor vision group. That is, participants were aged 8-83 years, and three children were aged under 15 years. Hence, the poor vision group was significantly older than the better vision group (Mann-Whitney U test, p < 0.01). The average observation periods were 54.3 ± 64.5 weeks in the better vision group and 115.5 ± 108.9 weeks in the poor vision group. Thus, the poor vision group had a significantly longer average observation period than the better vision group (Mann-Whitney U test, p < 0.01) (Table 1).

**Table 1:** Characteristics of the study

Total(n=59)	better visual acuity group	poor visual acuity group	P-value
Age	38.9±18.6（range; 5-70）	52.6±21.1（range; 8-83）	0.0066
Under 15 years old	5	3	uncountable
Average observation period(w)	54.3±64.5	115.5±108.9	0.0019
No.of female/total(%)	17/28(61)	20/31(65)	0.84
Recurrence	2	8	
Long optic nerve lesion on MRI/total(%)	13/26(50)	11/27(40)	0.41
Antibody			
anti-AQP4/anti-MOG/no positive(people)	1/3/25	0/4/27	uncountable

**Figure 2:**
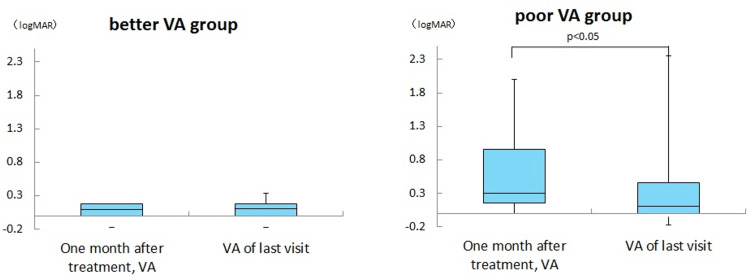
Comparison of visual acuity after one month of treatment and at the last visit in each group here was an improvement in visual acuity in the poor vision group between the visual acuity one month after the start of treatment and the last visit. Mann-Whitney U test, p < 0.05.

**Figure 3:**
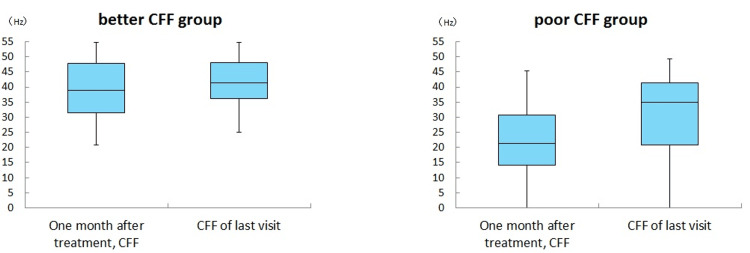
Comparison of CFF after one month of treatment and at the last visit in each group There was no significant difference between the CFF at one month after the start of treatment and CFF during the last visit in the better and poor vision groups.

There was an improvement in visual acuity in the poor vision group between the visual acuity one month after the start of treatment and the last visit. (Mann-Whitney U test, p < 0.05). There was no significant difference between the CFF at one month after the start of treatment and CFF during the last visit in the better and poor vision groups (Figures 2, 3).

**Figure 4:**
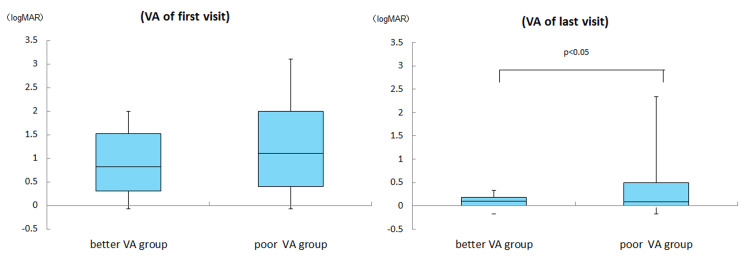
Comparison of visual acuity between initial and last visit There was no significant difference in visual acuity at the first visit between the two groups (Mann-Whitney U test, p=0.29). Visual acuity at the last visit was significantly less improved in the poor vision group at one month of treatment (Mann-Whitney U test, p < 0.05).

**Figure 5:**
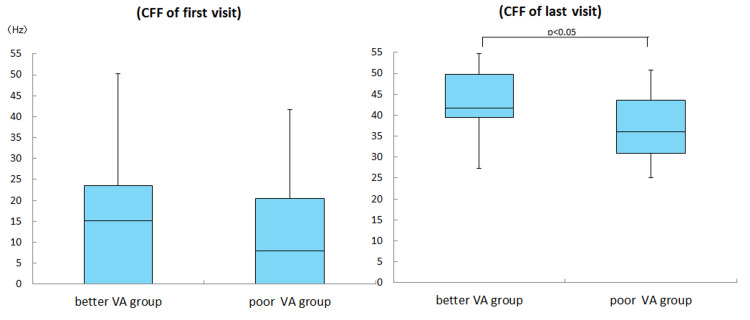
Comparison of CFF between initial and last visit There was no significant difference in CFF at the first visit between the two groups (Mann-Whitney U test, p=0.80). CFF at the last visit also showed significantly less improvement in the poor vision group at one month of treatment (Mann-Whitney U test, p<0.05).

The initial visual acuity (LogMAR) values were 0.9 ± 0.7 in the better vision group and 1.2 ± 0.9 in the poor vision group (Mann-Whitney U test, p = 0.29). The CFFs at the initial examination were 14.8 ± 17.3 Hz in the better vision group and 12.4 ± 14.2 Hz in the poor vision group. Hence, the two groups did not significantly differ in terms of CFF (Mann-Whitney U test, p = 0.80). The visual acuity values during the last visit (LogMAR) were -0.06 ± 0.1 in the better vision group and 0.4 ± 0.7 in the poor vision group. Thus, the poor vision group had a significantly lower improvement in visual acuity than the good vision group (Mann-Whitney U test, p < 0.05). The CFFs during the last visit were 41.1 ± 8.2 in the good vision group and 29.9 ± 14.9 in the poor vision group. Therefore, the poor vision group had a significantly poorer improvement in the CFF than the good vision group (Mann-Whitney U test, p < 0.05) (Figures 4, 5).

## Discussion

In this study, the poor vision group did not show a significant difference in visual acuity at the initial visit compared to the better vision group one month after the start of treatment for acute optic neuritis. However, their visual acuity was poor during the last visit. The poor vision group had a lower CFF recovery than the better vision group.

Visual acuity and CFF

The ONTT reported that poor initial visual acuity is the only prognostic factor of acute optic neuritis [5]. In this study, the poor visual acuity group had a poorer final visual acuity than the better visual acuity group one month after treatment for acute optic neuritis. This result was similar to that of the ONTT. CFF was not considered a prognostic factor in the ONTT. However, the current study found that even if the CFF one month after treatment was poor, the CFF value during the last visit did not significantly differ from that of the group with a better CFF. Hence, CFF can be recovered if vision improves.

Jacobson et al. have reported that the axons of the retinal ganglion cells can have a possible effect during recovery from optic neuritis. The parvocellular and magnocellular systems of retinal ganglion cells are involved in the damage in CFF [9].

Nakao et al. showed that patients with acute optic neuritis had decreased CFF and visual acuity upon diagnosis. However, there is a visual-CFF dissociation. The CFF remains low despite rapid improvement in visual acuity with steroid pulse therapy. Moreover, patients with residual dissociation are at risk of relapse and are often left with visual functional disability [10,11]. Nevertheless, further clinical studies on this notion should be performed.

Lin et al. evaluated the temporal course of CFF values after the onset of optic neuritis. Results showed that all patients with visual acuity below 0.3 LogMAR immediately after disease development had abnormal CFF values (10.9 ± 7.9 Hz). However, the visual acuity of some patients recovered to 0 LogMAR or better, and the CFF improved to 32.7 ± 5.1 Hz within the next 30 days. However, in 37% of the cases, the CFF remained abnormal [12]. In the current study, 16% of the patients with a final visual acuity of 0 LogMAR or better had abnormal CFF. This rate is higher than that previously reported.

In a multicenter optic neuritis trial conducted by Nakao in Japan, the post-treatment visual acuity-CFF dissociation was shorter in the steroid pulse group than in the oral mecobalamin group. The normal rate was significantly better in the steroid pulse group after 12 weeks of treatment [13]. All patients in this study were treated with steroid pulse therapy, which might have increased the rate of CFF improvement. However, Nakao reported that the normal CFF rate after one year of treatment further improved in both the steroid pulse group and the mecobalamin group. The significant difference disappeared. Therefore, if this study had a longer follow-up period, the improvement rate could have been higher.

Prognostic factors

In this study, the poor vision group was significantly older than the good vision group. The mean age at onset of anti-AQP4 antibody-positive optic neuritis was higher than at onset of anti-MOG antibody-positive or antibody-negative optic neuritis [14]. According to Waters et al., the sensitivities of ELISA and CBA for measuring anti-AQP4 antibodies were 60% and 73%, respectively [15]. Therefore, in patients with poor visual acuity at one month after treatment and elderly individuals, it may be advisable to consider treatment with anti-AQP4 antibody-positive optic neuritis, which includes reperforming the ELISA test and measurement using the CBA method, which can obtain false-negative results even if the ELISA test finding is negative.

Study limitations

The current study had several limitations. That is, patients could not be observed for an extended period. The longest follow-up period in the most protracted case was 480 weeks. However, there were several cases in which the follow-up period was shorter, and it varied considerably from point to case. Although steroid pulse therapy was a prerequisite for treatment, it cannot be denied that the details varied per patient. In particular, some patients received multiple sessions of steroid pulse therapy and others plasma exchange.

As for the antibody concentrations, not all patients were tested for each antibody. Therefore, the number of patients who tested positive for each antibody was small, and it could not be denied that some patients who were not tested might have also tested positive for the antibodies. In addition, there were no clear criteria for determining the performance of MRI measurements or contrast-enhanced MRI. Therefore, this notion should be evaluated in future studies.

## Conclusions

Visual acuity at the first visit did not affect the last visual acuity in either the better or poor vision group. Patients with good visual acuity one month after the start of treatment for acute optic neuritis also had better final visual acuity and CFF. This may reflect more damage to the optic nerve in the poor vision group. Therefore, visual acuity values one month after treatment initiation may be a predictor of treatment outcome.
